# Pulse sequence design for high field NMR with NV centers in dipolarly coupled samples

**DOI:** 10.1038/s41598-025-15899-5

**Published:** 2025-08-22

**Authors:** Carlos Munuera-Javaloy, Ander Tobalina, Jorge Casanova

**Affiliations:** 1 Department of Physical Chemistry, University of the Basque Country UPV/EHU, Apartado 644, 48080 Bilbao, Spain; 2 EHU Quantum Center, University of the Basque Country UPV/EHU, Leioa, Spain; 3 Institut für Theoretische Physik und IQST, Universität Ulm, Albert-Einstein-Allee 11, 89081 Ulm, Germany; 4 Department of Applied Mathematics, University of the Basque Country UPV/EHU, 01006 Vitoria-Gasteiz, Spain; 5 Arquimea Research Center, 38320 La Laguna, Spain

**Keywords:** Quantum information, Quantum metrology

## Abstract

Diamond-based quantum sensors have enabled high-resolution NMR spectroscopy at the microscale in scenarios where fast molecular motion averages out dipolar interactions among target nuclei. However, in samples with low-diffusion, ubiquitous dipolar couplings challenge the extraction of relevant spectroscopic information. In this work we present a protocol that enables the scanning of nuclear spins in dipolarly-coupled samples at high magnetic fields with a sensor based on nitrogen vacancy (NV) ensembles. Our protocol is based on the synchronized delivery of radio frequency (RF) and microwave (MW) radiation to eliminate couplings among nuclei in the scanned sample and to efficiently extract target energy-shifts from the sample’s magnetization dynamics. In addition, the method is designed to operate at high magnetic fields leading to a larger sample thermal polarization, thus to an increased NMR signal. The precision of our method is ultimately limited by the coherence time of the sample, allowing for accurate identification of relevant energy shifts in solid-state systems.

## Introduction

Over the past decade, quantum sensing–a notably prolific branch of quantum technologies^1,2^, has produced magnetometers able to detect ever weaker fields. Such development has had a deep impact in the realm of nuclear magnetic resonance (NMR)^3^, a field that, despite its unquestionable success, has limitations due to the inherent weakness of target signals necessitates the scanning of millimeter-sized samples. Nitrogen vacancy (NV) centers in diamond^4^, however, reported the detection of signals from smaller samples leading to NMR experiments with unprecedented spatial resolution. NV centers stand out for their capacity to operate at room temperature leading to smaller, and easier to operate magnetometers compared to platforms that require stringent conditions such as, e.g., superconducting quantum interference devices (SQUIDs).

NMR spectroscopy reveals frequency shifts relative to a base Larmor frequency which encode structural information about sample molecules as well as of their surrounding environment. In this scenario, one of the most impressive results produced by NV based NMR sensors –enabled by heterodyne protocols that overcome the resolution boundary posed by the coherence time of the NV centers^5,6^– is the record of spectral features from picoliter volume samples with high-resolution^7^. Nevertheless, in thermally polarized samples, these protocols require a high nuclear spin concentration (typically, highly protonated samples) and a significant number of repetitions to obtain meaningful results. A possibility for detecting samples at lower concentrations and achieving more competitive protocols is to hyperpolarize the samples in a previous step^8,9^. In addition, performing the experiment at high fields directly provides higher polarization rates while it facilitates the extraction of relevant information from the recorded spectra, as chemical shifts increase and J-couplings become clearer^10^. Although NV-NMR spectroscopy at high fields has not yet been experimentally demonstrated, recent proposals have introduced protocols that enable the acquisition of high-resolution spectra in strong external magnetic fields^11–13^. In particular, our proposal AERIS^12^ operates encoding the target nuclear energy shifts in the amplitude variation of the sample’s longitudinal magnetization which oscillates at a tunable rate –i.e., at a slow rate of, typically, tens of KHz even at large fields– during consecutive detections.

An important limitation of state-of-the-art NV-NMR spectroscopy is caused by the strong dipolar coupling among target nuclei, which results in intricate spectra that challenge data interpretation. This becomes especially critical in solid-state material research, where homonuclear dipole-dipole interactions hinder subtler couplings (heteronuclear interactions are less challenging as they can be eliminated by driving only one of the species). The NMR community has dedicated significant efforts to mitigate the impact of dipolar interactions^15^, and today solid-state NMR spectroscopy is extensively used in distinct research areas: In pharmaceutics it characterizes active pharmaceutical ingredients (APIs) and their interaction with excipients (inactive substances added to a drug that serve various purposes such as binding or preserving the API)^16–18^, among many other applications (see^19^ for an extensive review on the topic). In epidemiology, it provides key insights of the structure of molecules related with diseases as present in our societies as Alzheimer^20^. In energy storage research is used to characterize the local structure of solid materials used in batteries and fuel cells^21^.

These areas, along with many others, would largely benefit from sensors able to produce narrower spectral lines from smaller solid-state samples, especially over material surfaces where conventional NMR techniques are highly constrained. While NV-NMR spectroscopy emerges as the prominent technique to access samples in the microscale regime, it is currently limited to liquid state samples where dipole-dipole interactions get naturally averaged out due to fast molecular motion, leaving a plethora of solid-state applications out of its range of action. Extending high-field NV-NMR protocols, such as AERIS, to solid-state samples by effectively decoupling dipolar interactions, would unlock these applications^22,23^.

In this work, we devise a protocol that overcomes these limitations and enables high resolution NV-NMR spectroscopy of single crystals with strong homonuclear dipolar coupling at elevated external magnetic fields, allowing to take advantage from the higher polarization rates and stronger chemical shifts. Our protocol features the delivery of two radiation channels –radio-frequency (RF) and microwave (MW)– synchronized with measurements on an NV ensemble magnetometer. The RF channel drives the sample with a twofold purpose. On the one hand it effectively decouples the target nuclear spins, diminishing the effect of strong homonuclear couplings in the recorded spectra, and enabling the obtention of nuclear energy shifts. Remarkably, the decoupling benefits from increasing RF intensities could be specially effective in the small volume regime, where the driving field tends to be more homogeneous. In this regime, current RF antenna designs have demonstrated nuclear spin rotation rates in the tens to hundreds of kilohertz range^24,25^. On the other hand, the RF bridges the interaction among NV sensors and fast rotating nuclear spin by generating a slow-frequency NMR signal trackable by the NV sensor. Simultaneously, the MW channel delivers a tailored pulse sequence to the NV ensemble enabling the detection of the magnetic field emitted by the driven sample. This sequence is interspersed with measurements of the sensor’s state to construct the spectra in a heterodyne frame leading to a spectrum only limited by the nuclear sample coherence. Finally, we provide analytical expressions that map the detected resonances with target energy shifts.

## Methods

### RF modulation of nuclear spins

Lee and Goldburg (LG) showed in a seminal paper^26^ that an off-resonant continuous RF field cancels, up to first order, the contribution to the nuclear spin dynamics of homonuclear dipole-dipole interactions if the *LG condition* $\Delta =\pm \Omega /\sqrt{2}$ holds. Here, $\Delta = \omega _L-\omega _d$ is the detuning between the carrier frequency of the RF driving field ($\omega _d$) and the Larmor precession of the spins ($\omega _L$), and $\Omega$ is the Rabi frequency of the RF driving. Subjecting a spin ensemble to an off-resonant RF field leads to collective nuclear spin rotations along an axis tilted with respect to $\hat{z}$ (the direction of the static magnetic field). More specifically, *the tilted axis* –in the following *P*– has a component $\frac{\Delta }{\sqrt{ \Omega ^2 + \Delta ^2}}$ along $\hat{z}$, while its projection on the orthogonal *xy* plane is $\frac{\Omega }{\sqrt{ \Omega ^2 + \Delta ^2}}$.

**Figure 1:**
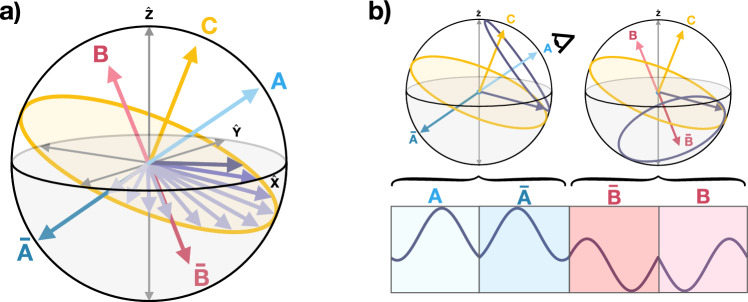
(**a**) Relative positions of axis $A$ (clear-blue), $\bar{A}$ (dark-blue), $B$ (clear-magenta), $\bar{B}$ (dark-magenta). The motion of the magnetization in between LG4 blocks ($A\bar{A}\bar{B} B$) is shown in purple. This evolution is described as a rotation in the plane (in yellow) perpendicular to the $C$ axis in accordance with the effective Hamiltonian in Eq. (4) for a single $\delta _i^*$. (**b**) (Top) Magnetization rotations during each of the four RF drivings. (Bottom) Projection of the magnetization onto $\hat{z}$ as it rotates during a full LG4 block. This projection determines the magnetic signal for the NV ensemble sensor.

Further developments have built upon the original LG sequence demonstrating the ability to remove higher order contributions of the dipole-dipole interaction, thus leading to even narrower spectral lines. Prominent examples are the frequency-switched (FSLG)^28^ and phase-modulated (PMLG)^29^ versions of the original LG sequence. Our protocol incorporates the advanced LG4 sequence^30^ over nuclei, which exhibits remarkable decoupling rates and enhanced robustness against RF control errors. The LG4 consists on concatenated blocks of four consecutive off-resonant RF drivings, all complying with the LG condition, leading to rotations along four different axes. This is, the rotation axis *P* alternates among $A,\bar{A},\bar{B}$ and $B$, whose relative positions are illustrated in Fig. 1a. Note that, at each block, nuclear spins undergo two sets of complementary rotations along axis pointing in opposite directions ($A, \bar{A}$ and $B, \bar{B}$). To further illustrate the journey of the magnetization during an LG4 block we include an animation^31^.

The nuclear spin Hamiltonian during each individual rotation of the LG4 sequence reads (see Supplementary Information^27^)

1 $$\begin{aligned} H = \sum _{i=1}^N\left( \frac{\pm \delta _i}{\sqrt{3}} + \bar{\Omega }\right) I^i_P, \end{aligned}$$

where $\delta _i$ is the *target nuclear shift* of the *i*th spin (its sign depends on the direction of the rotation, positive value $``+\delta _i''$ is assigned to rotations along *A* and *B*, and the negative value $``-\delta _i''$ to rotation along $\bar{A}$ and $\bar{B}$), $\bar{\Omega }=\sqrt{\Delta ^2+\Omega ^2}$ is the effective Rabi frequency, and the spin operator $I^i_P$ takes one of the following forms

2 $$\begin{aligned} I^i_A= & \left( \Omega I^i_x\sin {\alpha }+\Omega I^i_y\cos {\alpha }+ \Delta I^i_z\right) /\bar{\Omega }, \nonumber \\ I^i_{\bar{A}}= & - I^i_A,\nonumber \\ I^i_B= & \left( -\Omega I^i_x\sin {\alpha }+\Omega I^i_y\cos {\alpha }+ \Delta I^i_z \right) /\bar{\Omega },\nonumber \\ I^i_{\bar{B}}= & - I^i_B. \end{aligned}$$

According to the LG4 scheme^30^, the phase of the driving is set to $\alpha =55^\circ$ to minimize the line-width of the resonances.

Note that in Eq. (1) we assume that the internuclear interaction Hamiltonian $H_\textrm{nn}=\sum _{i>j}^N \frac{\mu _0\gamma ^2_n \hbar }{4\pi r_{i,j}^3} \bigg [\vec {I}_i \cdot \vec {I}_j - 3 (\vec {I}_j \cdot \hat{r}_{i,k}) (\vec {I}_j \cdot \hat{r}_{i,j})\bigg ]$ can be neglected due to the introduced decoupling sequence. This assumption simplifies the subsequent analysis. However, $H_\textrm{nn}$ will be taken into account in the numerical model in the results section.

In the remainder of this section, we analyze the signal emitted by the sample subjected to the RF decoupling fields and develop analytical expressions for the target energy shifts.

The magnetic field that originates from the sample during the nuclear spin rotation produced by each RF field of the LG4 follows the general form

3 $$\begin{aligned} s(t) = \Gamma \cos {(\bar{\Omega }t+\phi )}+b. \end{aligned}$$

Hereafter, we often refer to *s*(*t*) as the signal, as it constitutes the target field for the NV ensemble sensor. In fact, its amplitude $\Gamma$, phase $\phi$, and static bias *b* depend on the configuration of the nuclear spin ensemble and thereby on the $\delta _i$ energy shifts (see^27^), so detecting and properly reading *s*(*t*) enables to unravel the desired information.

Consequently, the LG4 meets a twofold goal. Namely: (i) It results in a nuclear spin dynamics with minimal effect from the dipole-dipole interaction (see^27^for the full derivation of the Hamiltonian in Eq. (1)), enabling the identification of the weaker but interesting $\delta _i$ shifts. (ii) It induces a tunable rotation speed in the sample ($\propto \bar{\Omega }$, see Eq. (3)), facilitating the interaction between nuclear spins and the NV ensemble sensor even at high external magnetic fields. Regarding point (ii), it is important to note that without using RF drivings on the sample, standard techniques based on imprinting in the NVs a rotation speed comparable to the nuclear Larmor frequency would necessitate the application of unrealistic MW fields. For context, in a magnetic field of approximately 2.35 Tesla, hydrogen spins rotate at a speed of $(2\pi )\times 100$ MHz, producing a signal hardly trackable by an NV ensemble sensor operating with conventional methods^7–9^.

Now, we examine the effects of RF decoupling fields in greater detail. Each RF driving (leading to the rotations along $A, \bar{A}$, $B, \bar{B}$) is applied for an interval $T = 1/\bar{\Omega }$. Consequently, the total signal emitted by the sample is a composite of distinct sinusoidal functions, condensed in Eq. (3), each persisting for a duration *T*. Figure 1b presents an illustrative example of *s*(*t*) by showing the rotation of a single magnetization vector (associated with a specific $\delta _i$) around axes $A, \bar{A}, B$ and $\bar{B}$.

Interestingly, with this RF control, the nuclear spins governed by Eq. (1) would perform a complete turn at each RF driving, constantly returning to their initial configuration if it were not for the $\delta _i$ shifts. These shifts slightly alter the nuclear spin state (i.e., the sample magnetization), thus imprinting a slower motion within the sample. More specifically, the sample magnetization at the end of each LG4 block is determined by a set of energy shifts $\delta ^*_j$ (distinct from $\delta _i$) according to the effective Hamiltonian:

4 $$\begin{aligned} H_\textrm{eff} = \sum _i \delta ^*_i I^i_C, \end{aligned}$$

where $I^i_C$ is a spin operator along an axis $C$ that bisects $A$ and $B$, see Fig. 1a, while

5 $$\begin{aligned} \delta _i^* = \delta _i \frac{\sqrt{1 + 2 \cos ^2{\alpha }}}{3}. \end{aligned}$$

In summary, this section demonstrates that each LG4 block alters the sample magnetization $\vec {M}$ through rotations along the $C$ axis, as depicted in Fig. 1a. An animation of the magnetization precession around the $C$ axis (leading to the yellow rotation plane Fig. 1a) is available in^32^. Moreover, we elucidate the mechanism governing the evolution of $\vec {M}$ through the effective Hamiltonian outlined in Eq.$(4)$, while Eq. (5) establishes analytical expressions connecting the rates of the effective rotations, $\delta ^*_i$, with the target nuclear shifts $\delta _i$.

In the next section we outline the protocol to monitor this effective precessions with the NV ensemble sensor and extract the desired $\delta _i$ energies from its recordings.

### Harvesting nuclear spin parameters with the NV ensemble

#### Geometrical interpretation of the phase accumulation

**Figure 2:**
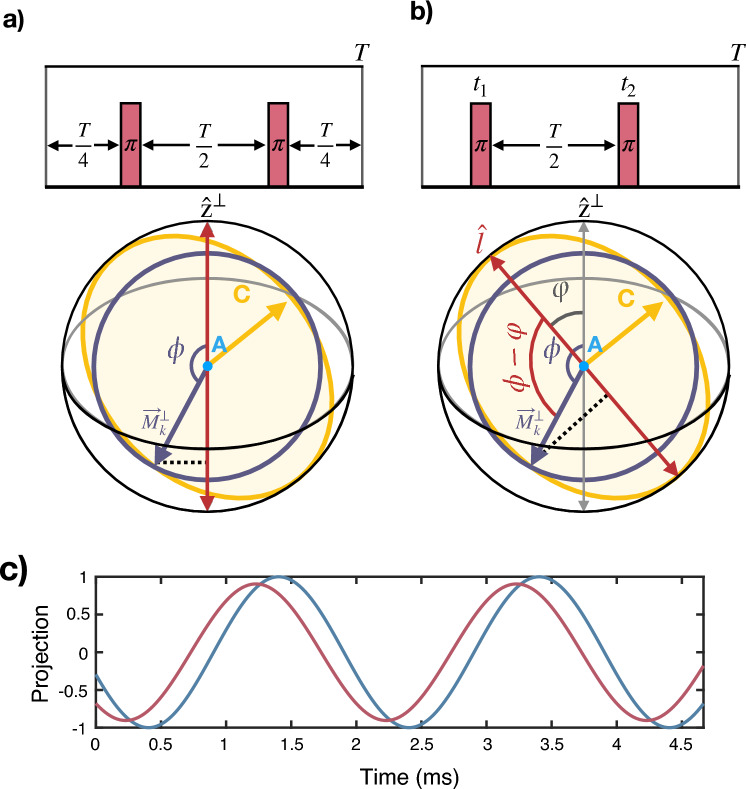
(**a**) Illustration of a CPMG pulse block (upper panel) and its geometric interpretation (lower panel). This panel shows a projection of the sphere in Fig. 1 (**b**) viewed in a direction parallel to axis $A$, as represented with the eye symbol. This view facilitates the representation of the projections onto the plane perpendicular to A of (i) The magnetization vector, denoted as $\vec {M}_k^\perp$, and (ii) The $\hat{z}$ axis, referred to as $\hat{z}^{\perp }$. In addition, it shows the trajectory followed by a magnetization vector during a rotation around *A* (purple circle) and after successive LG4 blocks (yellow ellipse). (**b**) Upper panel, pulse block of our tailored sequence where the initial $\pi$ pulse is delivered at a time $t_1$. With this control, the phase accumulation of the NV is proportional to the projection of $\vec {M}_k^\perp$ onto $\hat{l}$ (shown in red), an axis tilted away from $\hat{z}^\perp$ and aligned with the major axis of the yellow ellipse for optimal contrast. (**c**) Evolution of the projection of $\vec {M}_k^\perp$ onto axis $\hat{z}^\perp$ (red) and onto axis $\hat{l}$ (blue). The amplitude of the projection onto $\hat{l}$, resulting from the sequence in panel (**b**), reaches the maximum value of 1. As the phase accumulated by the NV is directly proportional to this projection, the timing of the pulses in (**b**) ensures the maximum phase accumulation amplitude.

The target magnetic field over the NV ensemble sensor is a concatenation of the sinusoidal signals in Eq. (3) (see lower panel in Fig. 1b). A particular RF field at the $k^\text {th}$ LG4 block (note that, the accumulative character of the rotations imposed by Eq. (4) make it crucial to identify the number of the block from now on), produces a nuclear spin rotation around a certain axis ($A, \bar{A}, B$ or $\bar{B}$) where the amplitude $\Gamma _k$ of the resulting signal $s_k(t) = \Gamma _k\cos {(\bar{\Omega }t+\phi _k)}+b_k$ is directly proportional to $\vec {M}_k^\perp$ (i.e., to the magnetization component which is orthogonal to the rotation axis –$A, \bar{A}, B$ or $\bar{B}$– at the start of each RF driving), and the phase $\phi _k$ corresponds to the angle between $\vec {M}_k^\perp$ and $\hat{z}^\perp$. The latter is the component of $\hat{z}$ that lies on the plane perpendicular to the rotation axis. See the lower panel in Fig. 2a and^27^ for more details.

We now use this geometric description to analyze the phase accumulated by each NV in the ensemble sensor when subjected to a generic pulse sequence. For this analysis, we choose a standard Carr-Purcell-Meiboom-Gill (CPMG) sequence^33,34^. In order to keep the discussion accessible, we focus on the signal produced by the nuclear spins rotating around $A$ and limit ourselves to an scenario involving a single effective energy shift, $\delta _i^*$. Note, however, that the following results and the consequent conclusions are valid for signals produced by nuclear spins rotating around axes $B, \bar{A}$ and $\bar{B}$ and in situations involving multiple shifts.

The phase accumulated by an NV center interacting with the signal $s_k(t)$ and subjected to the CPMG sequence reads (see^27^)

6 $$\begin{aligned} \Phi = \frac{4 |\gamma _e|}{\bar{\Omega }}\Gamma _k \cos {\left( \phi _k\right) }. \end{aligned}$$

Hence, the phase accumulated by each NV is proportional to the projection of $\vec {M}_k^\perp$ onto $\hat{z}^\perp$, or, in other words, to the quantity $\Gamma _k \cos {\left( \phi _k\right) }$. The lower panel of Fig. 2a provides a clarifying (probably most needed) graphic explanation.

With this description in mind we can summarize the phase acquisition stage as follows: The response of the NV centers to the signal emitted by the sample is determined by the initial sample magnetization. As the protocol advances, the magnetization vector precesses around *C* with an angular velocity $\delta ^*_i$ as described by Eq. (4). In the orthogonal plane with respect to *A*, this precession translates into an elliptical motion of the vector $\vec {M}_k^\perp$, shown as a yellow ellipse in Fig. 2a. Thus, the projection of $\vec {M}_k^\perp$ onto the $\hat{z}^\perp$ axis, and consequently the phase accumulated by the NV in successive blocks of the LG4, follow a sinusoidal function with frequency $\delta ^*_i$. The resulting expected value of the $\sigma _z$ operator of each NV in the ensemble at the $k^\text {th}$ LG4 block (after applying a final $\pi /2$ pulse to transform accumulated phase into populations), generalized to every $\delta _i^*$, reads:

7 $$\begin{aligned} \langle \sigma _z\rangle _k \approx 3 D_0 \sum _i \rho _i\cos {\left( \frac{4\delta _i^* k}{\bar{\Omega }} + \nu _0\right) }, \end{aligned}$$

where $\rho _i$ is the spin density of the *i*th nucleus, $D_0$ is detemined by the pulse sequence and $\nu _0$ depends on both the pulse sequence and the initial nuclear state. A formal derivation of (7) as well as further details can be found in^27^. The 0 subindex in Eq. (7) indicate that all parameters correspond to the reference CPMG sequence (note that, in the next section we derive an improved sequence). Thus, the NV response enclosed in Eq. (7) consists on a sum of sinusoidal functions that encode the different $\delta _i^*$ which can be then extracted via standard Fourier transform. Finally, $\delta _i$ targets can be obtained via a direct application of Eq. (5).

#### Sensing MW pulse sequence

With the geometrical understating developed in the previous section, now we present a tailored MW sequence to optimally detect the target $\delta _i$ shifts. This sequence retains a CPMG-like structure composed by two $\pi$ pulses spaced by *T*/2 to mitigates noise effects, *T* being the CPMG block length. Nonetheless, we adjust the timing of the pulses, see Fig. 2b, in particular the time at which the first pulse is applied ($t_1$ hereafter). Consequently, the phase accumulated by each NV in the ensemble sensor (recall that we are focusing on the signal produced by the nuclear spins rotating around *A*) reads

8 $$\begin{aligned} \Phi = \frac{4\Gamma _k|\gamma _e|}{\bar{\Omega }}\cos {\left( \phi _k-\varphi \right) }. \end{aligned}$$

In our geometrical framework, adjusting the timing of the pulses results in an accumulated phase proportional to the projection of $\vec {M}_k^\perp$ onto an axis $\hat{l}$, which is tilted at an angle $\varphi = \frac{\pi }{2} - \bar{\Omega } t_1$ relative to $\hat{z}^\perp$ (see Fig. 2b).

The ability to pivot the axis in which $\textbf{M}_k^\perp$ gets projected (note this can be done by selecting distinct values for $t_1$ since $\varphi = \frac{\pi }{2} - \bar{\Omega } t_1$) allows us to design a pulse sequence that maximizes contrast in the recorded spectra. From block to block, $\vec {M}_k^\perp$ evolves following an ellipse, therefore, we design the pulse sequence so that the phase accumulated by each NV is proportional to the projection of $\vec {M}_k^\perp$ into the major axis of the elipse. By doing so, the projecting axis and the direction that contains the extreme points of the elliptic path of $\vec {M}_k^\perp$ match, thereby yielding the maximum amplitude in the oscillation of $\Phi$ in successive blocks, see Fig. 2b. In particular, this is achieved by setting $\varphi = \arccos {\frac{\sqrt{3}\cos {\alpha }}{\sqrt{2 + \cos {2\alpha }}}}$, which determines the timing of the pulses as $t_{1,A}\approx 0.14\, T$ for optimal detection of the signal produced by the nuclear spins rotating around *A*. Repeating the same analysis for the signals produced by nuclear spin rotations around $\bar{A}$ and $\bar{B}$ we find $t_{1, {\bar{A}}} = \frac{T}{2}-t_{1, A}$ and $t_{1, {\bar{B}}} = t_{1, A}$.

**Figure 3:**
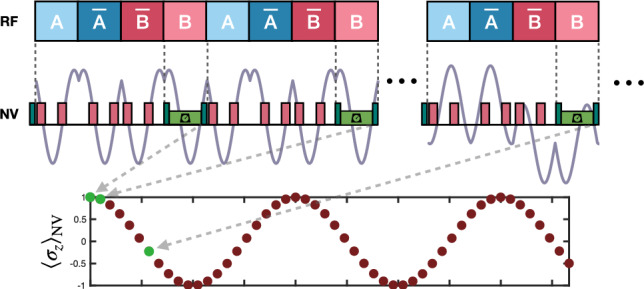
(Top) General layout of our protocol containing the RF control over nuclear spins and the MW pulse sequence on the NV ensemble. The magnetic field emitted by the nuclei as a consequence of the RF rotations is depicted in light purple. As time progresses, this field changes its shape, which changes the phase accumulated by the NV, while MW pulses keep always the same structure. (Bottom) Evolution of the expected results for the measurement over the NVs as the experiments progresses, showing a sinusoidal pattern with frequency $\delta ^*$. In the presence of additional shifts, the measurement outcomes evolve as a sum of sinusoidal components with corresponding frequencies $\delta ^*_i$, which can be extracted through Fourier transform analysis.

Summing up, our tailored MW pulse sequence is separated in blocks. Each block contains two $\pi$ pulses specifically timed to optimally detect the signal produced by the corresponding RF field. To maintain synchrony between the two control channels (MW and RF) and to avoid turning off the nuclear decoupling field, the sensor is measured and reinitialized while the RF field is on. Figure 3 shows the general layout of our protocol, including the drivings over the sample and the sensor, and showing the evolution of the expected outcomes in successive measurements, which read

9 $$\begin{aligned} \langle \sigma _z\rangle _k \approx 3 D_\textrm{opt} \sum _i \rho _i\cos {\left( \frac{4\delta _i^* k}{\bar{\Omega }} + \nu _\textrm{opt}\right) }, \end{aligned}$$

where $D_\textrm{opt}\approx 1.1 D_0$ (i.e., with the tailored MW sequence the contrast increases a $10\%$) and $\nu _\textrm{opt} = 0$ which corresponds to a initial sample magnetization oriented along the axis perpendicular to the $A$ and $B$ axes, achieved by a RF pulse that triggers the protocol. Finally, we access the effective frequencies $\delta _i^*$ by Fourier transforming the recorded data and obtain the target $\delta _i$ shifts from Eq. (5).

## Results

We test our protocol by simulating its implementation to detect the chemical shifts of hydrogen nuclear spins in ethanol molecules ($\mathrm{C_2H_6O}$) at high external magnetic field. Although ethanol typically exists as a liquid, we employ its solid configuration as an example of an ordered sample with strong homonuclear dipole-dipole couplings. In particular, ethanol molecules exhibit dipolar couplings of up to 17 kHz (the proton attached to the oxygen shows limited dipole interaction with the rest of the system so we exclude it from the simulations). High external fields improve nuclear magnetic resonance procedures, not only because it yields higher polarization rates, but also because it enhances the weaker, and thus harder to detect, energy shifts. In our simulations, we consider an external field of $B_0 = 2.1$ T, with chemical shifts of 3.66 ppm for three of the protons and 1.19 ppm for the other two, corresponding to frequency shifts of approximately 327 Hz and 106 Hz, respectively.

Our numerical simulation unfolds in two phases. First we find the target magnetic signal by simulating the evolution of the nuclear spin sample subjected to the LG4 decoupling sequence. The approximate Hamiltonian in Eq. (1) facilitates a deeper understanding of the nuclear dynamics and in particular the development of the geometrical interpretation that has set the ground for the design of our protocol. Our simulations, however, make use of the exact nuclear spin Hamiltonian, which reads

10 $$\begin{aligned} \begin{aligned} H(t)&= \sum _{i = 1}^{N = 5} \left\{ -\gamma _h \delta _i B_0 I_z^i + \left[ \Omega +\eta (t)\right] I_\phi ^i + \Delta I_z^i \right\} \\&\quad + \sum _{i > {j = 1}}^{N = 5}-\frac{\mu _0 \hbar \gamma _n^2}{8 \pi |r^{ij}|^3}\left( 3 r^{ij}_z-1\right) \left[ 3 I_z^i I_z^j - \vec {I}_i\cdot \vec {I}_j\right] , \end{aligned} \end{aligned}$$

where $\delta _i$ is the target nuclear shift of the *i*th hydrogen atom and $\eta (t)$ is the driving noise modeled with an Ornstein-Uhlenbeck process with a 1 ms correlation time and 0.24% amplitude.

We assume the sample starting in a completely mixed state. A triggering RF pulse sets the desired initial state $\rho (0)$, a thermal state oriented along the axis perpendicular to $A$ and $B$. From there, the evolution of the nuclear density matrix is simulated via the master equation

11 $$\begin{aligned} \dot{\rho } = -i\left[ H, \rho \right] + \frac{1}{2 T^*_2}\sum _{j = 1}^{N = 5}\left( 4 {I}^j_z \rho {I}^j_z-\rho \right) , \end{aligned}$$

where $T^*_2 = 0.2$ s is chosen to approximate the intrinsic coherence time in the absence of dipolar couplings (which are explicitly included in the Hamiltonian). This equation leads to a signal computed as

12 $$\begin{aligned} s(t) = \frac{\gamma _h \hbar \mu _0 \sigma _h g}{4\pi } \operatorname {Tr}\left[ \rho (t)\bar{I_z}\right] , \end{aligned}$$

where $\sigma _h = 5.2\times 10^{28} \mathrm {m^{-3}}$ is the number density of hydrogen spins for ethanol, and $g \approx ~4.1$ is a geometric factor that relates the sample magnetization and the magnetic field in the NV location, see^7,12^ for further details.

**Figure 4:**
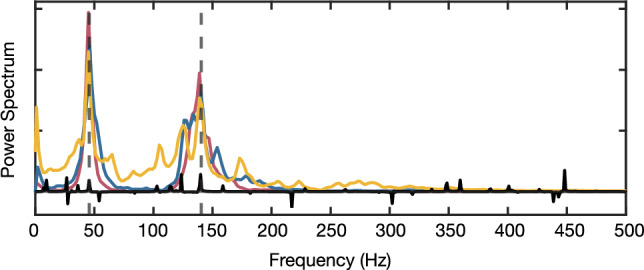
Spectra obtained from three simulations with RF Rabi frequencies of $(2\pi )\times$100 kHz (yellow line), $(2\pi )\times$150 kHz (blue line), and $(2\pi )\times$200 kHz (red line). Vertical dashed gray lines indicate the expected resonances, i.e. the two $\delta ^*_i$, of approximately 140 Hz and 45 Hz. For comparison, a spectrum obtained with AERIS is depicted (black line), using an RF Rabi frequency of $(2\pi )\times$150 KHz. In all simulations, the nuclear sample starts in a thermal state corresponding to a temperature of T=300 K in an external magnetic field of 2.1 T. The sample evolves for a total time of 0.5 s.

In the second phase, we simulate the evolution of the NV ensemble interacting with *s*(*t*) and subjected to the sensing MW pulse sequence of our protocol. The Hamiltonian that governs the dynamics of each NV centers reads

13 $$\begin{aligned} H = \gamma _e s(t)\frac{\sigma _z}{2}+C(t)\frac{\sigma _z}{2}+\frac{\Omega _\textrm{NV}(t) \sigma _\phi }{2}, \end{aligned}$$

with $\Omega _{\text {NV}}(t)$ the control Rabi frequency, and $C(t)\frac{\sigma _z}{2}$ describing potential RF-induced crosstalk on the NVs. Following the protocol devised in this work, they interact with the signals produced by the nuclear spin rotations around axis $A, \bar{A}$ and $\bar{B}$ and accumulate a phase determined by the state of the sample magnetization. During the time that corresponds to the delivery of the $B$ RF field over the sample, we transform the accumulated phase into a population difference with a $\pi /2$ pulse and simulate the measurement, after which the sensor is reinitialized and the protocol repeats. Finally, a Fourier transform of the measurements provides the spectra displayed in Fig. 4, which proves the ability of our protocol to access the chemical shifts of the molecule.

Figure 4 shows three different experiments with increasing RF intensities. As expected, stronger RF drivings lead to clearer spectra, less distorted by spurious peaks which arise due to incomplete averaging of dipolar couplings. In particular we simulate RF drivings with Rabi frequencies of $(2\pi )\times$100 kHz, $(2\pi )\times$150 kHz, and $(2\pi )\times$200 kHz, all attainable values by state of the art antennas^24,25^, and set the Rabi frequency of the MW control at 20 MHz in all cases. Note that when the RF Rabi frequency exceeds the 17 KHz dipolar coupling strength by an order of magnitude, the resulting spectra become significantly cleaner and more resolved. As intended, our method leads to resonance peaks centred in $\delta _i^*$ from which one can extract the target nuclear shifts $\delta _i$ using Eq. (5). For comparison, we include the results of a fourth simulation using AERIS^12^, which does not incorporate any dipolar coupling suppression technique. In this case, the obtained spectra (black-curve) is distorted as a consequence of the strong nuclear dipolar couplings.

In summary, we have demonstrated that our protocol effectively identifies the target energy shifts $\delta _i$ with strong nuclear dipole-dipole interactions at high external magnetic fields.

## Conclusions

We have designed a protocol that utilizes LG4 sequences and a tailored NV pulse train to identify chemical shifts in the presence of strong dipole-dipole interactions. The RF field serves two key purposes: (i) decoupling nuclear spins and (ii) generating a nuclear signal oscillating at a moderate frequency that can be measured by the NVs, allowing the protocol to operate at high magnetic fields. By incorporating a tailored MW sequence on the NV for signal detection, we achieve effective retrieval of chemical shifts. Finally, the accuracy of our method is ultimately limited by the nuclear sample decoherence, thus surpassing the limitations imposed by NVs dephasing and thermalization. Our findings pave the way for the advancement of microscale NMR techniques and broaden their application in diverse fields, such as materials science, chemistry, and biology.

## Supplementary Information

Supplementary Information 1.

Supplementary Information 2.

Supplementary Information 3.

Supplementary Information 4.

## Data Availability

The code used to produce the results in this study is available at https://github.com/carlosmunueraj/NV-LG4-DD-samples.
