# Supplementary material for: Pulse sequence design for high field NMR with NV centers in dipolarly coupled samples
Source: Sci Rep. 2025 Aug 22;15:30956. doi: 10.1038/s41598-025-15899-5 (PMC12373868; doi:10.1038/s41598-025-15899-5)
Supplement: Supplementary file 1 — Supplementary Information 1. [file 41598_2025_15899_MOESM1_ESM.pdf]

Video material legend:

Supplementary video 1:
Trajectory of the magnetization during a single LG4 sequence.

Supplementary video 2:
Precession of the magnetization during many LG4 sequences, showing the global precession around the C axis.
