# Supplementary material for: Pulse sequence design for high field NMR with NV centers in dipolarly coupled samples
Source: Sci Rep. 2025 Aug 22;15:30956. doi: 10.1038/s41598-025-15899-5 (PMC12373868; doi:10.1038/s41598-025-15899-5)
Supplement: Supplementary file 4 — Supplementary Information 4. [file 41598_2025_15899_MOESM4_ESM.pdf]

Supplemental Material for:
Pulse Sequence Design for High Field NMR with NV Centers in Dipolarly Coupled Samples

I. NUCLEAR SPIN DYNAMICS UNDER RF DRIVINGS

Under an RF field (that will rotate nuclei around the $A, \bar{A}, B$, or $\bar{B}$ axes) the nuclear spin Hamiltonian including dipole-dipole terms among nuclear spins reads

$$H = \sum_i \left[\gamma_n B_z I_z^i + \delta_j I_z^i + 2\Omega I_x^i \sin(\omega_d t - \alpha)\right] + \sum_{i>j} \frac{\mu_0 \gamma_n^2 \hbar}{4\pi r_{i,j}^3}\left[\vec{I}_i \cdot \vec{I}_j - 3(\vec{I}_i \cdot \hat{r}_{i,j})(\vec{I}_j \cdot \hat{r}_{i,j})\right], \tag{S1}$$

where $r_{i,j}$ is the distance between each pair of nuclei ($\hat{r}_{i,j}$ its a unitary vector such that $\vec{r}_{i,j} = r_{i,j}\hat{r}_{i,j}$), γ_N is the nuclear gyromagnetic ratio, μ_0 is the vacuum permeability, $\vec{I}_i = \left(I_x^i, I_y^i, I_z^i\right)$ are the nuclear spin operators for the ith spin, Ω and ω_d are the Rabi and carrier frequencies of the radio-frequency (RF), α is a tunable phase of the RF, and δ_i represents the deviation (i.e. the energy shift) of the ith nuclear spin from the Larmor precession rate $\omega_L = \gamma_n B_z$ owing to its particular magnetic environment. The accurate determination of δ_j is the target of the sensing protocol introduced here.

In a rotating frame w.r.t. $(\omega_L - \Delta)\sum_i I_z^i$, and under the secular approximation of the dipole-dipole term that eliminates fast rotating terms by invoking the rotating wave approximation, Eq. (S1) simplifies to

$$H = \sum_i \left[(\Delta + \delta_i) I_z^i + \Omega I_\alpha^i\right] + \sum_{i>j} \frac{\mu_0 \gamma_n^2 \hbar}{4\pi r_{i,j}^3}\left[1 - 3\left(r_z^{i,j}\right)^2\right]\left[I_z^i I_z^j - \frac{1}{2}(I_\alpha^i I_\alpha^j + I_{\alpha^\perp}^i I_{\alpha^\perp}^j)\right]. \tag{S2}$$

where $I_\alpha^i = \left(I_x^i \sin\alpha + I_y^i \cos\alpha\right)$, and $I_\alpha^{i\,\perp} = \left(I_x^i \cos\alpha - I_y^i \sin\alpha\right)$.

Introducing a new spin basis, defined by rotating the original axes around $\alpha^\perp$,

$$\begin{aligned} I_P^j &= \cos(\theta) I_z^j + \sin(\theta) I_\alpha^j, \\ I_Q^j &= \cos(\theta) I_\alpha^j - \sin(\theta) I_z^j, \\ I_{Q^\perp}^j &= I_{\alpha^\perp}^j, \end{aligned} \tag{S3}$$

and defining the rotation angle through $\cos\theta = \frac{\Delta}{\sqrt{\Omega^2+\Delta^2}}$, and $\sin\theta = \frac{\Omega}{\sqrt{\Omega^2+\Delta^2}}$, allows to rewrite the sample Hamiltonian as

$$\begin{aligned} H = \bar{\Omega}\sum_i I_P^i + \sum_i \delta_i[\cos(\theta) I_P^i - \sin(\theta) I_Q^i] + \sum_{i>j} \frac{\mu_0 \gamma_n^2 \hbar}{4\pi r_{i,j}^3}\left[1 - 3(r_{i,j}^z)^2\right]\Big\{ \cos^2(\theta) I_P^i I_P^j + \sin^2(\theta) I_Q^i I_Q^j \\ - \cos(\theta)\sin(\theta)(I_P^i I_Q^j + I_Q^i I_P^j) - \frac{1}{2}\Big[\cos^2(\theta) I_Q^i I_Q^j + \sin^2(\theta) I_P^i I_P^j + \cos(\theta)\sin(\theta)(I_P^i I_Q^j + I_Q^i I_P^j) + I_{Q^\perp}^i I_{Q^\perp}^j\Big]\Big\}, \end{aligned} \tag{S4}$$

where the effective rotation rate around I_P reads $\bar{\Omega} = \sqrt{\Delta^2 + \Omega^2}$. Finally, many terms can be neglected by a secular approximation with respect to $\bar{\Omega}\sum_i I_P^i$. The remaining terms in the dipolar interaction disappear *magically* when the angle that defines the change of basis in Eq. (S3) satisfies $\cos(\theta) = \pm 1/\sqrt{3}$, or, equivalently, when the Lee-Goldburg condition $\Delta = \pm\Omega/\sqrt{2}$ is met, leading to

$$H = \sum_{i=1}^{N}\left(\frac{\pm\delta_i}{\sqrt{3}} + \bar{\Omega}\right) I_P^i, \tag{S5}$$

where $\pm\delta_j/\sqrt{3}$ are the parallel components of the shifts with respect to the effective rotation axis P, and its sign is the same as the sign of Δ. Note that any combination of Ω and Δ that complies with the Lee-Goldburg condition produces the described decoupling effect. In particular, for a given intensity of the RF field, this can be detuned from the top and from the bottom with respect to the Larmor. Moreover, the previous derivation is valid for any phase α of the RF field. This freedom has been exploited to develop more elaborated control schemes that concatenate various RF fields, such as the LG4 sequence implemented in our protocol.

In the LG4 sequence, each driving axis is applied during a time $T = 2\pi/\bar{\Omega}$ following the order A, $\bar{A}$, $\bar{B}$, and B (see main text). In order to obtain the effective dynamics of a full LG4 block, we write the explicit propagator

$$U_{\mathrm{LG4}} = U_B U_{\bar{B}} U_{\bar{A}} U_A = e^{-i\sum_{i=1}^{N}\left(\frac{\delta_i}{\sqrt{3}}+\bar{\Omega}\right)I_B^i T} e^{-i\sum_{i=1}^{N}\left(-\frac{\delta_i}{\sqrt{3}}+\bar{\Omega}\right)I_{\bar{B}}^i T} e^{-i\sum_{i=1}^{N}\left(-\frac{\delta_i}{\sqrt{3}}+\bar{\Omega}\right)I_{\bar{A}}^i T} e^{-i\sum_{i=1}^{N}\left(\frac{\delta_i}{\sqrt{3}}+\bar{\Omega}\right)I_A^i T}. \tag{S6}$$

In every propagator, we can do the following change

$$e^{-i\sum_{i=1}^{N}\left(\pm\frac{\delta_i}{\sqrt{3}}+\bar{\Omega}\right)I_P^i T} = e^{-i\sum_{i=1}^{N}\pm\frac{\delta_i}{\sqrt{3}}I_P^i T} e^{-i\sum_{i=1}^{N}\bar{\Omega}I_P^i T} = e^{-i\sum_{i=1}^{N}\pm\frac{\delta_i}{\sqrt{3}}I_P^i T}, \tag{S7}$$

where we used that $e^{-i\sum_{i=1}^{N}\bar{\Omega}I_P^i T} = e^{-i\sum_{i=1}^{N}2\pi I_P^i} = \mathbb{I}$. Assuming that $\bar{\Omega} >> \pm\frac{\delta_i}{\sqrt{3}}$, we can Trotterize the LG4 propagator to obtain

$$e^{-i\sum_{i=1}^{N}\left(\frac{\delta_i}{\sqrt{3}}I_B^i - \frac{\delta_i}{\sqrt{3}}I_{\bar{B}}^i - \frac{\delta_i}{\sqrt{3}}I_{\bar{A}}^i + \frac{\delta_i}{\sqrt{3}}I_A^i\right)T}. \tag{S8}$$

Finally, substituting the expression for each axis operator of Eq. (2) in the main text, we obtain the propagator

$$e^{-i\sum_{i=1}^{N}\left[\frac{\delta_i}{\sqrt{3}\Omega}\left(\Omega I_y^i\cos\alpha+\Delta I_z^i\right)\right]4T}. \tag{S9}$$

From this expression, we reach the final effective Hamiltonian after rearranging the terms

$$H_{\mathrm{eff}} = \sum_i \delta_i^* I_C^i, \tag{S10}$$

where $I_C^i = \frac{\sqrt{2}I_y^i\cos\alpha+I_z^i}{\sqrt{2\cos^2\alpha+1}}$ and $\delta_i^* = \delta_i\frac{\sqrt{1+2\cos^2\alpha}}{3}$.

II. ACCUMULATED PHASE

As stated in the main text, the signals received by the NV adhere to a general form Eq. (3) in the main text. When a two pulse CPMG sequence is applied on the NV sensor (see Fig. (1) in the main text) with π pulses applied at times t_1 and t_2, the phase accumulated by the NV at stage k is:

$$\Phi_k = \int_0^{t_1}\left[|\gamma_e|\Gamma_k\cos\left(\bar{\Omega}t+\phi_k\right)+b_k\right]dt - \int_{t_1}^{t_2}\left[|\gamma_e|\Gamma_k\cos\left(\bar{\Omega}t+\phi_k\right)+b_k\right]dt + \int_{t_2}^{T}\left[|\gamma_e|\Gamma_k\cos\left(\bar{\Omega}t+\phi_k\right)+b_k\right]dt, \tag{S11}$$

where we choose the separation of both pulses to be $\frac{T}{2}$, which ensures the cancellation of the static b_0 term

$$\Phi_k = \frac{2|\gamma_e|\Gamma_k}{\bar{\Omega}}\left[\sin(\bar{\Omega}t_1+\phi_k) - \sin(\bar{\Omega}t_2+\phi_k)\right] = \frac{4|\gamma_e|\Gamma_k}{\bar{\Omega}}\cos(\phi_k-\varphi), \tag{S12}$$

with $\varphi = \frac{\pi}{2} - \bar{\Omega}t_1$.

As the sample evolves under the LG4 sequence, the amplitude Γ_k and phase ϕ_k of the NMR signal evolve, see Fig. (**??**) (a, b). If the signal gets projected about some axis, e.g. $\Gamma_k\cos\phi_k$, the variation of this projection is a simple sinusoidal function (see Fig. (**??**) (c)) which is exactly what we need in order to extract the information using a discrete Fourier transform. This result can be understood geometrically, see main text.

Once we choose a projection angle axis, we can compute the adequate timing for the CPMG sequence as

$$\varphi = \varphi_{\mathrm{opt}} \rightarrow \varphi_{\mathrm{opt}} = \frac{\pi}{2} - \bar{\Omega}t_1 \rightarrow t_1 = \frac{\pi}{2\bar{\Omega}} - \frac{\varphi_{\mathrm{opt}}}{\bar{\Omega}}. \tag{S13}$$

For optimal pulse positions, we select the angle matching the major axis of the ellipse. This axis is orthogonal to both $\hat{A}$ and $\hat{C}$, i.e., $\left(0, -\frac{1}{\sqrt{2+\cos 2\alpha}}, \frac{\sqrt{2}\cos\alpha}{\sqrt{2+\cos 2\alpha}}\right)$. Then, the angle θ_A is measured with respect to the orthogonal component of $\hat{z}$ concerning $\hat{A}$. This angle is:

$$\varphi_{\mathrm{opt}} = \arccos\frac{\sqrt{3}\cos\alpha}{\sqrt{2+\cos 2\alpha}}. \tag{S14}$$

III. ANALYTICAL EXPRESSION

Here we provide details of the derivation of the analytical expression for the expected value of the measurements performed with the NV. Our starting point is the fact that the NV will couple to a signal proportional to the $\hat{z}$ component of the sample magnetization.

Focusing on the kth driving stage around $\hat{A}$, we can describe the expected signal as:

$$s \propto \hat{M}(t) \cdot \hat{z} = \hat{M}(t)\left(\hat{z}^{\perp} \sin\theta_{\rm LG} + \hat{A}\cos\theta_{\rm LG}\right), \tag{S15}$$

where $\hat{M}(t)$ is the magnetization vector and the $\hat{z}$ axis was split in the parallel and perpendicular components with respect to axis $\hat{A}$, and $\theta_{\rm LG} = \arccos\frac{1}{\sqrt{3}}$ is the magic angle. We can describe the time dependency of the magnetization during the driving stage A by employing the Rodrigues' rotation formula as

$$\hat{M}(t) = \hat{M}_k \cos\bar{\Omega}t + \left(\hat{A}\times\hat{M}_k\right)\sin\bar{\Omega}t + \hat{A}\left(\hat{A}\cdot\hat{M}_k\right)\left(1-\cos\bar{\Omega}t\right), \tag{S16}$$

where $\hat{M}_k$ is the magnetization vector at the beginning of the kth sequence. Substituting in Eq. (S15), we get

$$s \propto \left[\hat{M}_k\cdot\hat{z}^{\perp}\cos\bar{\Omega}t + \left(\hat{A}\times\hat{M}_k\right)\cdot\hat{z}^{\perp}\sin\bar{\Omega}t\right]\sin\theta_{\rm LG} + \hat{A}\cdot\hat{M}_k\cos\theta_{\rm LG}. \tag{S17}$$

We can now split the magnetization vector into its parallel and perpendicular components with respect to $\hat{A}$ as $\hat{M}_k = \left(\vec{M}_k^{\parallel} + \vec{M}_k^{\perp}\right)$. With this we reach expression

$$s \propto |\vec{M}_k^{\perp}|\sin\theta_{\rm LG}\cos\left(\bar{\Omega}t+\phi\right) + |\vec{M}_k^{\parallel}|\cos\theta_{\rm LG}, \tag{S18}$$

where ϕ is the angle between $\vec{M}_k^{\perp}$ and $\hat{z}^{\perp}$. Notice how this expression exactly matches the shape of Eq. (3) in the main text.

Substituting in Eq. (8) of the main text, we obtain

$$\Phi \propto -\frac{4\gamma_e\sin\theta_{\rm LG}}{\bar{\Omega}}|\vec{M}_k^{\perp}|\cos(\phi-\varphi) = -\frac{4\gamma_e\sin\theta_{\rm LG}}{\bar{\Omega}}\hat{M}_k\cdot\hat{l} \tag{S19}$$

with $\hat{l}$ a vector perpendicular to $\hat{A}$ and tilted φ with respect to $\hat{z}^{\perp}$.

We can now generalize to all the driving stages by describing the precession motion of the initial magnetization vectors employing Rodrigues' formula once again

$$\hat{M}_k = \hat{M}_0\cos\left(\frac{4\delta_i^*k}{\bar{\Omega}}\right) + \left(\hat{C}\times\hat{M}_0\right)\sin\left(\frac{4\delta_i^*k}{\bar{\Omega}}\right) + \hat{C}\left(\hat{C}\cdot\hat{M}_0\right)\left[1-\cos\left(\frac{4\delta_i^*k}{\bar{\Omega}}\right)\right]. \tag{S20}$$

Starting with an initial magnetization $\hat{M}_0$ in the orthogonal plane with respect to $\hat{C}$ and an angle μ with respect to $\hat{x}$ (which resides in this plane), and including factors for the signal amplitude, we obtain the formula for the accumulated phase

$$\Phi_k = D_\varphi\rho_i\cos\left(\frac{4\delta_i^*k}{\bar{\Omega}} + \mu - \beta_\varphi\right), \tag{S21}$$

where $D_\varphi = \frac{-\gamma_e\hbar^2\gamma_h^2\mu_0 g\sin(\theta_{\rm LG})}{8\bar{\Omega}\pi^2k_BT}\sqrt{\left(\frac{\cos\varphi\sin\alpha}{\sqrt{3}} - \cos\alpha\sin\varphi\right)^2 + \frac{\left(\sqrt{3}\cos\alpha\cos\varphi+\sin\alpha\sin\varphi\right)^2}{2+\cos(2\alpha)}}$, ρ_i is the spin density of the ith nucleus, and $\beta_\varphi = \arctan\frac{3\left(\sqrt{3}\cos\alpha\cos\varphi+\sin\alpha\sin\varphi\right)}{\sqrt{2+\cos(2\alpha)}\left(\sqrt{3}\cos\varphi\sin\alpha-3\cos\alpha\sin\varphi\right)}$. Here, g is a geometric factor that relates the sample geometry with the signal amplitude in the NV site, k_B is the Boltzmann constant, and T is the temperature. See [S1, S2] for further details on the signal amplitude expression. It can be checked that $\varphi_{\rm opt}$ does indeed maximize D_φ. The total accumulated phase of the three drivings A, $\bar{A}$, B is simply $3\Phi_k$, provided that $\nu_\varphi = \mu - \beta_\varphi$ is the same in the three stages, which in our case we choose to add up to 0.

Finally, to consider all effective chemical shifts δ_i^* it suffices to sum all the contributions. Assuming a small angle Φ_k, the final formula for the expected value of σ_z is

$$\langle\sigma_z\rangle_k \approx 3D_\varphi\sum_i\left[\rho_i\cos\left(\frac{4\delta_i^*k}{\bar{\Omega}} + \nu_\varphi\right)\right], \tag{S22}$$

which gives us the desired spectrum upon Fourier transform.

[S1] D. R. Glenn, D. B. Bucher, J. Lee, M. D. Lukin, H. Park, and R. L. Walsworth, High-resolution magnetic resonance spectroscopy using a solid-state spin sensor, Nature **555**, 351 (2018).
[S2] C. Munuera-Javaloy, A. Tobalina, and J. Casanova, High-Resolution NMR Spectroscopy at Large Fields with Nitrogen Vacancy Centers, Phys. Rev. Lett. **130**, 133603 (2023).
